# Analytical and Chemometric Characterization of Sweet Pedro Ximénez Sherry Wine during Its Aging in a *Criaderas y Solera* System

**DOI:** 10.3390/foods12091911

**Published:** 2023-05-07

**Authors:** Manuel J. Valcárcel-Muñoz, María Guerrero-Chanivet, María del Carmen Rodríguez-Dodero, Daniel Butrón-Benítez, María de Valme García-Moreno, Dominico A. Guillén-Sánchez

**Affiliations:** 1 Bodegas Fundador S.L.U., C/San Ildefonso, n 3, 11403 Jerez de la Frontera, Spain; mjc.valcarcel@gmail.com (M.J.V.-M.); maria.guerreroch@uca.es (M.G.-C.); daniel.butron@uca.es (D.B.-B.); 2 Departamento de Química Analítica, Facultad de Ciencias, Instituto Investigación Vitivinícola y Agroalimentaria (IVAGRO), Campus Universitario de Puerto Real, Universidad de Cádiz, 11510 Puerto Real, Spain; maricarmen.dodero@uca.es (M.d.C.R.-D.); dominico.guillen@uca.es (D.A.G.-S.)

**Keywords:** raisin, aging, wood, multiple linear regression, sherry wine

## Abstract

Pedro Ximénez is a naturally sweet sherry wine produced in southern Spain from raisined Pedro Ximénez grape must and aged using a traditional *Criaderas y Solera* system. Complete analytical characterization has been useful in determining which parameters are the most influential in the aging of this wine. The organic acids, volatile compounds (higher alcohols, esters, aldehydes, and acetals), and phenolic compounds of this wine evolve during its aging, mainly through physico-chemical reactions and the contributions of wood compounds. During their aging, Pedro Ximénez sherry wines develop their organoleptic profiles, as tasting sessions have confirmed. A strong correlation between the aging of a wine and the parameters analyzed has also been corroborated through an MLR analysis. This allowed for the development of a model that, by using just 8 of the variables considered in the study, led to the determination of wine samples’ ages at over 97% confidence. This constitutes a rather useful tool for wineries to control Pedro Ximénez sherry wine aging processes.

## 1. Introduction

Amontillado, Fino, Oloroso, Palo Cortado, and certain natural sweet wines, such as Moscatel or Pedro Ximénez, are produced in the Jerez-Xérès-Sherry protected designation of origin (PDO). The dry fortified wines are made from Palomino grapes, while the natural sweet wines are made from their respective varieties of Moscatel or Pedro Ximénez grapes. Aging is carried out according to a traditional dynamic aging system from the sherry area known as *Criaderas y Solera*. All of this, along with the specific climate conditions in Marco de Jerez, make these wines a highly appreciated product by oenologists worldwide [1,2].

Pedro Ximénez sherry wine (PXSW) is a liqueur wine with a protected designation of origin (Jerez-Xérès-Sherry). Specifically, it is a natural sweet wine obtained by fortifying alcohol, including alcohol, distillate, or brandy, with overripe or raisined grape musts of the Pedro Ximenez variety, in addition to an oxidative aging process for at least two years [3]. The freshly harvested overripe grapes at over 13.5° Baumè are directly exposed to sunlight, on esparto grass or mesh nets in specifically appointed areas known as “*paseras*”, for a period of between 5 and 15 days, depending on weather conditions. Thus, partial dehydration and darkening of the berries take place, which result in raisins with a reducing sugar content over 450 g/L. The raisins are then pressed to obtain a very sweet must, which is then fortified with wine alcohol up to between 15 and 17% ABV (alcohol by volume) in order to stop the fermentation process [4,5,6]. Young Pedro Ximénez fortified wine (YPXFW) is aged in a winery through oxidative aging under a *Criaderas y Solera* system. It then acquires a more or less intense ebony color with iodized iridescences and a very dense visual appearance (it is probably the sweetest wine in the world) [4]. It is very complex in aromas, where notes of dried fruits, such as raisins, figs, or dates, stand out, together with others of honey, syrup, and candied fruits, with long-aged ones beginning to develop hints of toast (coffee, bitter chocolate, and cocoa) or liquorice. It possesses a natural acidity that comes from the raisined grapes, which balances its sweetness and makes of it a fresh, harmonious, velvety, and unctuous wine on the palate, with a very pleasant finish [4,5,6].

PXSW ages under a dynamic system of *Criaderas y Solera*, characteristic of sherry wines. This system consists of oak casks of 500–600 L of capacity, which are arranged at several scales or levels, according to the aging time of the wine that they contain [7,8].

The quality and botanical origin of the oak casks that contain aging PXSW, as well as the treatment and previous use that they have had, are of great relevance with regard to the aging process (these casks must have been previously seasoned before being used as part of a *Criaderas y Solera* system [9]). Casks, themselves, are not inert elements, but rather contribute with numerous compounds from their own compositions to aging wines and facilitate the occurrence of specific chemical reactions in them that have an impact on wines’ organoleptic properties [10,11,12]. Wood mainly contributes with phenolic compounds [13], especially ellagitannins and phenolic aldehydes derived from cinnamic acid and benzoic acid, such as coniferaldehyde, sinapaldehyde, vanillin, and syringaldehyde [13,14]. Additionally, furfurals and their derivatives, such as 5-methylfurfural and 5-hydroxymethylfurfural, are obtained from the wood [14]. Other substances that are transferred by the wood of casks into the wine during its aging are pentoses, polysaccharides, lactones, fatty acids, inorganic substances, and alcohols, which confer complexity and quality to the products [14].

In this work, complete physicochemical characterization of PXSWs aged through a *Criaderas y Solera* system as well as a sensory study of these wines with an average age of 40 years in the Solera have been carried out. A chemometric study has also been conducted in order to determine which variables might present any relevant correlations between the parameters considered and the aging times of the wines that would lead to the development of multiple linear regression (MLR) models that would allow for the estimation of the aging time of the PXSWs simply by identifying the most influential parameters involved in their aging processes. This model should represent a useful tool for wineries to regularly control the quality of their wines.

## 2. Materials and Methods

### 2.1. Samples

Bodegas Fundador, S.L.U. (Jerez de la Frontera, Cadiz, Spain) provided the PXSWs, the casks, the wine distillates for the fortification used, and the winery facilities where this study was conducted. The casks used in this study have been selected from the industrial *Criaderas y Solera* system at Bodegas Fundador, S.L.U, and are old enough for us to consider that the system of continuous mixtures on which the *Criaderas y Solera* systems are based have reached equilibrium on all scales. All of them were made of American oak (*Quercus alba*), with medium wood grain, medium-toasted, and 500 L of capacity.

**Figure 1:**
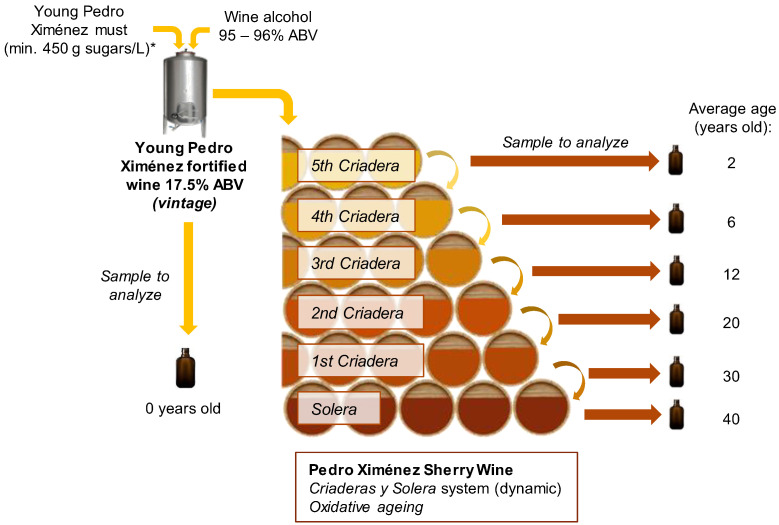
Diagram of the studied Pedro Ximénez sherry wines’ aging process and sampling. * Fermentation stopped via fortification.

Figure 1 displays the process diagram being studied. All wine replenishments for the different aging scales were carried out using the PXSW from the next younger aging scale previously fortified at 18% ABV, except for the 5th Criadera, which was replenished with YPXFW at 17.5% ABV.

This study lasted four years, and each wine category was sampled during the month of May of each year, as described below:

- Young Pedro Ximénez fortified wine (YPXFW) (vintage): Each year a combined sample was taken from 5 industrial 20,000 L tanks containing the young fortified wine from that year’s harvest. One liter was taken from each of the tanks and blended to obtain a final five-liter sample. Before proceeding with the sampling, the wine from the tanks was tasted to ensure that it presented no organoleptic flaws.
- Pedro Ximénez sherry wine (PXSW) aged in a *Criaderas y Solera* system: The system consisted of one Solera and five Criaderas that comprised 25 × 500 L casks per scale and involved the performing of regular removals, known as *sacas*, and replenishments, known as *rocíos*. The 5th Criadera was an average of 2 years old (YO); the 4th Criadera, 6 YO; the 3rd Criadera, 12 YO (PX12YO); the 2nd Criadera, 20 YO; the 1st Criadera, 30 YO; and the Solera, 40 YO (PX+30YO). Each year, over the whole 4-year research period, a combined 5 L sample was gathered by extracting 200 mL of wine from each of the 25 casks that made up each of the scales. Before proceeding with the sampling, the wines from each of the casks were tasted in order to verify that no organoleptic flaws were present.

A combined sample from each scale (6 different scales + YPXSW) taken over the 4 years of the study, i.e., 4 samples from each wine age, was physicochemically characterized. All the samples were analyzed in triplicate. Only the samples taken in the fourth year were used for the sensory evaluation.

### 2.2. Reagents

Ultrapure water (EMD Millipore, Bedford, MA, USA); UHPLC-grade acetone (VWR International, Radnor, PA, USA); and 0.1 M sulfuric acid (Sigma-Aldrich, Saint Louis, MO, USA) were used for the preparation of the eluent used for the analysis of organic acids.

Folin–Ciocalteau reagent and anhydrous sodium carbonate from Merck (Darmstadt, Germany), as well as ultrapure water (EMD Millipore, Bedford, MA, USA), were used to determine the Folin–Ciocalteau Index.

For the analysis of phenolic and furfural compounds, HPLC-grade acetonitrile, supplied by Panreac (Barcelona, Spain); acetic acid, supplied by Merck (Darmstadt, Germany); and ultrapure water, supplied by EMD Millipore (Bedford, MA, USA) were used to prepare the UHPLC phases.

Sigma-Aldrich (Saint Louis, MO, USA) supplied all of the reagents for the analysis of the oenological control parameters in addition to the calibration standards.

### 2.3. Parameters of Oenological Control

The official methodology described by the International Organisation of Vine and Wine (OIV) was used to determine the oenological control parameters: The density of wines (g/L) was directly measured by means of a DMA-5000 digital density meter (Anton Paar, Ashland, OR, USA). The alcoholic strengths (% ABV) were determined by distilling the wine and then measuring the distillate density [15] by means of a DMA-5000 digital density meter (Anton Paar, Ashland, OR, USA). The pH of wines was determined with a BASIC 20 pH meter (Crison Instruments SA, Barcelona, Spain). The total acidity of wines (g tartaric acid/L) was determined via potentiometric titrations at pH 7 [16]. The volatile acidity of wines (g acetic acid/L) was analyzed through the use of an AA3 HR Autoanalyzer segmented flow analyzer (Seal Analytical, Norderstedt Stadt, Germany) [17]. The glycerol content (g/L) was determined via an enzymatic method [18]. The total sulfur (mg/L) was analyzed through the use of the Ripper method [19]. The sulfate concentration (in g of K_2_SO_4_/L) was calculated via the gravimetry of BaSO_4_ precipitation [20]. The calcium, potassium, copper, and iron contents (mg/L) were determined via atomic absorption spectroscopy, using a PinAAcle 900F system equipped with the software application WinLab32 AA, both by Perkin Elmer (Boston, MA, USA). The total dry extract of wines (g/L) was determined via gravimetry, following the official standard procedure [21]. The reducing substances (g/L) were also analyzed through following the official standard procedure [22]. The sugar-free extract of wines (g/L) was determined according to the following formula [21]:

$$Sugar-free\;extract\left(\frac{g}{L}\right)=Total\;dry\;extract\left(\frac{g}{L}\right)-Reducing\;substances\left(\frac{g}{L}\right)$$

### 2.4. Organic Acids

A 930 Compact IC Flex, from Metrohm (Madrid, Spain), with a Metrosep Organic Acids column of 9 µm particle size and 250 × 7.8 mm (i.d.) [8], was employed to analyze citric, lactic, malic, succinic, and tartaric acids (mg/L). The software application used for data acquisition and processing was MagicNet 3.3 (Metrohm, Madrid, Spain). The compounds were identified via a comparison of their retention time against the standard used.

### 2.5. Higher Alcohols, Aldehydes, Methanol, and Ethyl Esters

An Agilent 7890B Gas Chromatograph (Agilent Technologies, Santa Clara, CA, USA), coupled with a flame ionization detector [8,23], was used to determine methanol, acetaldehyde, acetaldehyde-diethylacetal, esters (diethyl succinate, ethyl lactate, ethyl acetate, ethyl hexanoate, ethyl octanoate, ethyl decanoate, ethyl dodecanoate, and ethyl tetradecanoate), and higher alcohols (isobutanol, n-propanol, 2-methyl-1-butanol, 3-methyl-1-butanol, n-hexanol, and 2-phenylethanol). Standards were prepared in an ethanol/ultrapure water solution at the same alcoholic strength as the wines for the identification, based on their retention times, and quantification of the samples. For the ISTD (internal standard) for aldehydes and higher alcohols, 2-pentanol was used, and ethyl undecanoate was used for major esters. The samples were injected after distilling them at the same alcoholic strength. The results have been expressed as mg/L.

### 2.6. Folin–Ciocalteau Index

The Folin–Ciocalteau index (FCI) was used to determine the total content in phenolic compounds [24]. A Lambda 25 spectrophotometer from Perkin Elmer (Boston, MA, USA) was used to measure absorbance at 750 nm on glass cuvettes with a light path length of 10 mm. Previously, a calibration curve was performed with gallic acid in a concentration range between 0 mg/L and 750 mg/L. Gallic acid standards were prepared in a solution at the same alcoholic strength as the wines, containing 425 g/L of a mixture of glucose and fructose in a 0.93:1.00 ratio, similar to that of wines. The results were expressed as mg gallic acid equivalent (GAE)/L.

### 2.7. Phenolic Compounds and Furfurals

Fifteen phenolic compounds (caffeic acid, p-coumaric acid, ferulic acid, gallic acid, p-hydroxybenzoic acid, protocatechuic acid, syringic acid, vanillic acid, trans-caftaric acid, cis-p-coutaric acid, trans-p-coutaric acid, fertaric acid, p-hydroxybenzaldehyde, syringaldehyde, and vanillin) and two furfurals (5-hydroxymethylfurfural and furfural) were determined via UHPLC [23,25]. A Waters Acquity UPLC fitted with an Acquity UPLC C18 BEH column, 100 × 2.1 mm (i.d.) and 1.7 µm particle size, as well as a PDA detector (Waters Corporation, Milford, MA, USA) has been used. Nylon membranes with a pore size of 0.22 µm were used to filter the samples and standards. Standards were prepared in an ethanol/ultrapure water solution at the same alcoholic strength as the wines. The analyzed compounds were identified via a comparison of the retention time and the UV–Vis spectrum of the sample, as well as with the standard used. The results were expressed as mg/L [23,25].

### 2.8. Determination of the Brown Color of the Wines

The measure of the absorbance at 470 nm was used to determine the brown color of the wines [26]. All of the measurements were carried out through the use of a Perkin Elmer spectrophotometer—a Lambda 25 spectrophotometer (Perkin Elmer, Boston, MA, USA). All of the results were expressed as absorbance units. Prior dilutions were made via the use of a hydroalcoholic mixture at 17% ABV (deionized water and 96% ABV neutral wine alcohol) when necessary. The measurements were performed in triplicate.

### 2.9. Tasting Sessions

A properly isolated room, in order to allow the tasters to focus on their job, was used for the tasting sessions. Individual tastings sessions were carried out at an ambient temperature of 22 °C [27]. The tasting panel was made up of 7 tasters from Bodegas Fundador’s staff, and all of them had over 10 years’ experience in sherry wine tasting.

Two PXSWs of different average ages were evaluated in the tasting sessions: 3rd Criadera (12 years), named PX12YO, and Solera (40 years), named PX+30YO, given that the specifications for the Jerez-Xérès-Sherry designation of origin (DO) only allow the certification of 12-year-old wines and over 30-year-old wines (VORS—very old rare sherry), as well as YPXFWs.

For each wine, 50 mL was served in black wine-tasting glasses [28], and these were capped, for at least ten minutes, with a glass lid in order to stabilize their headspace prior to tasting. Nose and mouth perception data were recorded.

The definitions associated with the descriptors, selected according to the criteria of the DO tasting panel, are specified in Table 1. The olfactory–gustatory standard that corresponds to the maximum intensity of most of them but dried fruit (value: 9) on the 9-point interval numeric scale used [29] was a 50-year-old PXSW (special casks). A young vintage wine obtained from the must of sunlight-unexposed overripe Pedro Ximénez grapes, to which distilled alcohol at 95.4% ABV was added to stop its fermentation (17% ABV and 217 g sugars/L), was used as the minimum intensity standard (value: 1) except for the descriptor dried fruits, where it showed the highest intensity [29].

### 2.10. Statistical Analysis

The Statgraphics 19 software package (Statgraphics Technologies, Inc., The Plains, VA, USA) was employed for the multiple linear regression analysis, ANOVA, and Fisher’s least significant difference test. Microsoft Excel 2016 (Microsoft Corp., Redmond, WA, USA) was employed to process the rest of the statistical parameters.

Regarding the sensory data, the statistical analysis included an ANOVA, as well as a factorial analysis. The software application Statistica 8.0 (StatSoft Inc., Tulsa, OK, USA) was used for both of these analyses. Microsoft Excel 2016 (Microsoft Corp., Redmond, WA, USA) was used to generate a spider web chart.

## 3. Results and Discussion

### 3.1. Parameters of Oenological Control

#### 3.1.1. Alcoholic Strength

The alcoholic content of the PXSWs studied (Table 2) revealed a decreasing trend over their aging, where significant differences (ANOVA) were found corresponding to the different aging scales, since the high concentration of sugars in this wine prevents the staves that form the casks from tightly binding together and preventing ethanol losses via evaporation. This is due to the high density of this type of sweet wine as well as to the hygroscopicity of the sugars, which prevent the wine from sufficiently penetrating the wood pores and making them swell, as occurs in the case of the oxidative aging of dry fortified sherry wines (Amontillado, Oloroso, or Palo Cortado). In fact, in these cases, the trend is quite the opposite, with the alcohol content increasing as the wine ages [7,8,30,31]. These losses in ethanol and other volatiles, together with other minor losses attributable to the transpiration of the water molecules through the pores of the wood that form the casks containing the wine, is a phenomenon traditionally known as “*merma*” [8,32].

The average annual value corresponding to *merma* over the 4 years of the study was approximately 2.5% in the *Criaderas y Solera* system analyzed, i.e., a yearly reduction in the wine volume in the casks by 2.5%, which means an increment of the concentrations of the compounds present in the casks [7,8].

#### 3.1.2. Acidic Parameters

The ANOVA results do not reveal a clear trend in the wines’ pH values; significant differences were found between the total acidity corresponding to the different aging scales, as well as between their volatile acidity levels (Table 2).

A decrease in pH with aging (4.50–4.06) was observed in the PXSW *Criaderas y Solera* system (Table 2). Plastering is not used for the vinification of Pedro Ximénez, nor are corrections of the fixed acidity with tartaric acid at harvesting employed, which result in a higher pH and a lower total acidity in relation to other sherry wines where these practices are applied [7,8,30,33,34]. The stabilization of pH at around 4.0 in the PXSW from the 1st Criadera and from the Solera must be a consequence of the increased concentration caused by the *merma* of the acids from the raisined grapes and to the precipitation of insoluble salts that seek a chemical equilibrium by concentrating their ions (mainly potassium bitartrate and calcium tartrate).

The total acidity in the PXSW *Criaderas y Solera* system increases with the aging time (Table 2), from 2.95 g tartaric acid/L in the YPXFW to 5.25 g tartaric acid/L in the Solera PXSW. This is explained by several factors, such as the following: the increases in acetic acid content via the oxidation of the ethanol in the medium [23]; the concentration effect resulting from the *merma* of organic and inorganic acids in the wine; and the contributions of acidic substances from the wood (mainly acetic acid [14,35] and fatty acids [14]). This is the case despite the decrease in the concentration of tartaric acid that takes place in the wine during its aging as a consequence of the precipitation of its potassium and calcium salts.

Likewise, an increase in volatile acidity during aging is observed in these wines, from 0.52 g acetic acid/L in the YPXFW to 1.06 g acetic acid/L in the Solera PXSW (Table 2). This is due to the following factors: the oxidation of the alcohol in the medium [23]; the contributions of acetic acid from the wood [14]; and the effect of the *merma*. In addition, the volatile acidity of solely the Solera wine shows that the product has been aged correctly from a microbiological point of view (without bacterial infections); this value is much lower than 2.10 g acetic acid/L, which is the maximum value according to the specifications set out for the Jerez-Xérès-Sherry DO corresponding to this type of old wine [36].

#### 3.1.3. Glycerol

Significant differences (ANOVA) have been found in the glycerol content because of aging for all of the wines studied. The evolution of glycerol concentration was upward with a longer aging time (Table 2), and reached values close to 10 g/L in the Solera wine. The concentration of this compound is caused by the evaporation and/or transpiration associated with the *merma* process.

In wine, glycerol is mainly a secondary product of alcoholic fermentation [37], although it is also explained by the hydric stress suffered by grape cells during their raisining. This process triggers an intracellular anaerobic metabolic process that leads to the synthesis of glycerol [38], which acts as an osmolyte. In the young PXSW, despite the discontinuation of the fermentation process and because of the two effects mentioned above, some wines with contents around 3 g/L glycerol have been reported.

#### 3.1.4. Sulfates

Although the differences between the first and second scales with respect to sulfate content were not significant, as the aging time grew longer statistically significant differences (ANOVA) between the scales could be observed. The presence of sulfates is exclusively attributable to the grapes, but an increment was observed according to aging time (Table 2), mainly due to the effect of the *merma*. Nevertheless, although a greater rise has been expected, this did not take place, which could be due to precipitations, mainly of calcium sulfate [39].

#### 3.1.5. Potassium and Calcium

Significant differences (ANOVA) have been found in most of the wines studied between potassium and calcium contents as a function of aging [40,41]. In the younger scales of the aging system, a decrease in potassium and calcium content was observed (Table 2) with respect to the initial wine, while in the older wines their content increased due to the *merma*. This reduction in the younger wines was explained by the precipitation of inorganic and organic salts that takes place during their aging. In the wines’ hydroalcoholic medium, potassium bitartrate is supersaturated, which makes it precipitate during the aging in an attempt to achieve an equilibrium between potassium and tartaric acid. These precipitations are favored by the temperature drops that occur in cellars during the winter. In the case of calcium, it is the calcium salts of tartrate and sulfate that precipitate. In certain wines with a long aging period [40,41], oxalate also precipitates because of the higher-than-usual concentration that results from the *merma*.

#### 3.1.6. Total Sulfur Dioxide

Significant differences (ANOVA) could be observed between the values corresponding to total sulfur dioxide in the different aging scales, since no new sulfur was added after the crushing and sulfur oxidizes into sulfate. The values registered for the YPXFW were a consequence of the additions that take place during the crushing of the grapes (90–120 mg/L). On the other hand, during aging, a drop in the total sulfur content of the wine, which reached almost 0 mg/L in the Solera, was observed (Table 2).

#### 3.1.7. Density, Total Dry Extract, Reducing Substances, and Sugar-Free Extract

Significant variations (ANOVA) were observed between the levels of these parameters (total dry extract, reducing substances, and sugar-free extract) and the different aging scales.

The density value of PXSWs is much higher than that of other types of sherry wines [7,8,30], due to their high sugar contents. This parameter also evolved slightly upward during the aging studied, and significant discrepancies have been observed between the density values corresponding to the wines from the different aging scales.

During the aging, an increment in the content of soluble solids as well as an increasing density of the wines due to the *merma* were observed. The poor air tightness of the casks allows for alcohol losses that favor this upward tendency in soluble solids and density.

Total dry extract, reducing substances, and sugar-free extract are parameters that increased during the aging process of the PXSWs studied.

Thus, the total dry extract increased with aging due to the *merma* phenomenon, which affects all of the compounds involved in its quantification, i.e., acids, glycerol, polyphenols, and reducing substances, such as the hexoses, pentoses, and polysaccharides extracted from the wood or the glucose and fructose originating from the Pedro Ximénez raisins. PXSWs have much higher total dry extract values than the dry sherry wines [7,8,30] because of the greater amount of sugars in them.

With regard to the oxidative aging, the concentration of reducing substances as a consequence of the *merma* and also because of the contributions from the wood’s hemicellulose [13,14] increased with aging time. This parameter is mainly associated with the quantification of sugars and polysaccharides, whose content is greater in PXSWs than in other sherry wines because of the presence of the glucose and fructose originating from the raisins [7,8,30].

Sugar-free extract is a measure of all non-volatile substances at 110 °C. The origin of these substances can be varied, including the following: the raisins themselves; the different fermentation reactions that take place in the wine before it is fortified to stop the alcoholic fermentation process; the wood of the cask where the wine is aging; or the *merma* effect, among others. This analytical parameter rose with oxidative aging from 20.83 g/L in the YPXFW up to 31.03 g/L in the Solera wine.

### 3.2. Organic Acids

Significant differences (ANOVA) were observed between the concentrations of each organic acid depending on the scale in the system studied. The concentrations of lactic, malic, citric, succinic, and tartaric acids in PXSWs depend mainly on their content levels in the grapes themselves, i.e., on the anaerobic metabolic reactions that take place in the cells of the grape berries during their raisining process [42,43], as well as on the greater or lesser activity that yeasts and bacteria may have had between the crushing of the raisins and the fortification of the must, since the presence of these microorganisms in their metabolic pathways may decrease or increase in the wines [37]. Tartaric acid presented a tendency to decrease during the aging of the wine because of the insolubilization of its calcium and potassium salts [8,39]. The other acids increased their presence (Table 2) as a result of the *merma*.

The main sources of citric acid in YPXFW are raisins and stems [44]. The different acids in grape stems are usually salified. Citric acid, therefore, reaches the young wine through the pressing of the raisin bunches, where the presence of the stems enhances the extraction of the must from inside the raisins. It can be seen in Table 2 that, during the aging, there was an increment of citric acid from 210 to 385 mg/L.

A similar behavior is observed for malic acid, and its origin is also the raisined grapes [42,43]. In the absence of either any significant metabolic activity by bacteria or yeasts or precipitations in which this compound may intervene, it becomes the organic acid at the highest concentration in the oldest wines, reaching almost 2.3 g/L in the Solera (Table 2). The concentration of this acid increases during the aging process as a result of the *merma*.

The metabolic activity of yeasts during must fermentation produced succinic and lactic acids [37]; this is why their respective contents are not very high in PXSWs, where the fermentation process is halted just after it has started. A growing concentration, owing to the *merma*, is also observed as the wine is aged (Table 2); however, their levels at 514 mg succinic acid/L and at 653 mg lactic acid/L in the Solera wine are lower than those found in other Sherry wines, where fermentation is completed before they are aged under a similar oxidative aging system [7,8,37].

### 3.3. Volatile Substances

#### 3.3.1. Aldehydes, Methanol, and Higher Alcohols

Significant dissimilarities (ANOVA) between the different aging scales were observed with respect to these volatile compounds, except in those cases where no notable evolution was registered with the aging, such as n-hexanol.

Aldehyde content (acetaldehyde and its diethyl acetal) in YPXFW is not very high, and it depends on the amounts produced in the initial fermentation of the must, as well as on the amounts contributed by the 95.4% ABV wine distillate used for its fortification. Aldehyde content presents an upward trend during the aging (Table 3) as a result of alcohol oxidation; however, their content does not reach the expected levels in PXSWs, which may be explained by the poor air tightness of the casks in comparison to that of casks used to age dry sherry wines. This lack of tightness favors the loss of these highly volatile compounds through evaporation.

The methanol content present in YPXFW (Table 3) is in accordance with the degradation level of the grape cell walls that takes place during the raisining process. Methanol is thus released due to the demethoxylation of the pectins catalyzed by pectin esterases [41,45]; additionally, the high pressure used for the crushing of the raisins causes the extraction of precursors of this compound from the solid parts of the grape bunches. The volatile composition of the wine distillates used for the fortification of the must also plays a relevant role. The trend that has been observed during the aging process is not clearly defined, such that the content levels remain more or less steady, probably because of two mutually compensating effects: evaporation because of the poor air tightness of the casks and the *merma*.

All of the higher alcohols studied evolved upward during aging (Table 3), mainly due to the concentration of these compounds with the *merma* effect. Alcoholic fermentation produces higher alcohols via different metabolic pathways that correspond to the decarboxylation/deamination of amino acids [37,39], as well as from the smaller contribution from the 95.4% ABV wine distillate used for the fortification of YPXFWs. Since alcoholic fermentation is minimal in YPXFWs, the concentrations of n-propanol, isobutanol, 2-methyl-1-butanol-1, and 3-methyl-1-butanol are lower than in other oxidatively aged sherry wines [7,8,31] where the must is fully fermented.

The n-hexanol content was low in all of the wines (Table 3), ranging from 0.22 to 0.32 mg/L, and remained stable throughout their oxidative aging.

During alcoholic fermentation, 2-phenylethanol is a compound produced by the yeasts from 2-phenylalanine [45]. Furthermore, its content increases during the aging of the wine due to either the hydrolysis of 2-phenylethyl acetate or to the oxidation of ethanol and its subsequent condensation with the phenols from the wood [46], as well as to the *merma*. Its contents, shown in Table 3, are lower than those in other fully fermented and oxidatively aged sherry wines [7,8].

#### 3.3.2. Major Ethyl Esters

Significant differences (ANOVA) in major ethyl esters were observed depending on the system scales. Among the esters studied (Table 3), ethyl acetate stands out as one of the compounds that most evolves and in an increasing manner with the aging of the wines analyzed. In the case of PXSWs, acetic acid is produced via the oxidation of ethanol in the medium [23], and there is also a contribution of acetic acid from the casks’ wood [14]. Ethyl acetate is formed via the esterification of acetic acid with the ethanol in the medium, and it also increases its concentration because of the *merma*. Its maximum level is reached in the Solera wine at 129.0 mg/L, which is much lower than that registered for other oxidative aging Sherry wines after the same time in the aging casks [14]. This is partly due to the lower alcohol content of PXSWs compared to similarly aged dry sherry wines, which affects the equilibrium required to form the corresponding ester, but is also due to the aforementioned poorer tightness of the casks containing Pedro Ximénez wines.

Table 3 also shows the results corresponding to other ethyl esters. It should be noted that the esters studied belong to two families: The first one, the fatty acid ethyl esters (ethyl hexanoate, ethyl octanoate, ethyl decanoate, ethyl dodecanoate, and ethyl tetradecanoate), are partially produced by the esterification with ethanol of the fatty acids produced by yeasts during the glyceropyruvic fermentation of the must sugars and represent one of the most important groups of aromatic compounds that play a significant role regarding the organoleptic characteristics of wine [47]. These esters have also been detected in significant amounts in the musts from grapes that have undergone intracellular anaerobic metabolism (a process that is favored by grape raisining) [38]. The concentrations of these esters hardly reached 1 mg/L in the system analyzed, with ethyl octanoate and ethyl decanoate being the major ones. Moreover, only ethyl octanoate shows a growing trend with aging, which explains why no significant variations between the different aging scales could be observed in most cases. The concentration caused by the *merma* did not seem to affect the concentration of these esters in the old wines, since they are highly insoluble in media below 45% ABV. Regarding the contents of fatty acid ethyl esters, a similar behavior to that observed in other types of sherry wines was registered [7,8].

The second of the ester families are the ethyl esters of organic acids (ethyl lactate and diethyl succinate), which are generated via the chemical esterification of ethanol with their organic acids that come from the activity of the yeasts during the initial alcoholic fermentation that takes place in the must when making YPXFWs. Table 3 shows the concentrations measured in the *Criaderas y Solera* system and in the young wine. Thus, an increasing trend associated with aging time was observed (reaching 51.37 mg/L ethyl lactate and 11.17 mg/L diethyl succinate in the Solera).

### 3.4. Folin–Ciocalteau Index, Phenolic Composition, and Lignin-Derived Compounds

For the FCI and the phenols analyzed, significant differences (ANOVA) were found between the different aging scales. In the system studied, a constant increase in the FCI was observed with aging (Table 4). This was due to the transfer of compounds from the wood, the effect of *merma*, and the different chemical reactions that take place during the aging process, as well as those that involve the phenolic substances contributed by the grapes [14,48].

Total phenol contents are much higher in PXSWs than in the other sherry wines [7,8] because of the significant phenolic and furanic aldehyde concentrations resulting from the raisining of the grapes exposed to direct sunlight [4,5,6,49]. The PXSW Solera had an FCI of 2.871 mg GAE/L.

It was also observed that aging PXSWs (Table 4) contain certain lignin-derived compounds from oak wood, but their concentrations are lower than those found in dry sherry wines [7,8]. This may be due to the poorer penetration that these sweet wines achieved into the wood pores because of the high sugar contents and high viscosity in comparison to dry sherry wines. These, with a higher fluidity, as well as alcohol content, penetrate the wood more easily and achieve greater extractions of wood compounds.

Regarding the phenols that are specific to grapes (Table 4), it was observed that, as the aging time increases, the wines present a lower content of tartaric acid derivatives. This was possibly due to hydrolytic processes, since their free phenolic structure increased at the time of hydrolysis (p-coumaric acid, caffeic acid, and ferulic acid) [44], although a decrease in the contents of caffeic acid and ferulic acid was also observed later in lengthily aged wines, with the exception of p-coumaric acid. Furthermore, when analyzing the phenols derived from tartaric acid in the starting wines, it was noted that the sunlight exposure process undergone by Pedro Ximénez grapes caused hydrolysis and a decrease in their concentration in the YPXFWs, compared to those determined for wines of the Palomino variety [7]. Other phenols, such as gallic acid—contributed by the raisined grapes themselves as well as by the degradation of gallotannins yielded by the wood—or syringic acid, which comes in small quantities from the raisined grapes as well as, in greater amounts, from the wood lignin or the oxidation of its aldehyde (syringaldehyde) [13], increased their concentration as a consequence of the *merma* effect [50].

There are higher contents of p-hydroxybenzoic acid and protocatechuic acid in YPXFWs, because of the raisining effects [4,5,49], compared to dry sherry wines [7,8]. During aging, these contents remain at a high level and, in addition, they may increase both as a result of the *merma* and because of the contributions from the wood.

Table 4 shows a high content of furfurals (5-hydroxymethylfurfural and furfural) in YPXFWs. These high values may be attributable to the Maillard reactions that occur during the heating of grape berries as they are directly exposed to sunlight to obtain the raisins. Moreover, a sugar medium with a pH of between 4 and 5 promotes these reactions [51]. As the wine was aged under an oxidative process in a *Criaderas y Solera* system, a significant increase in 5-hydroxymethylfurfural and furfural content was observed, again attributable to Maillard reactions, where the long aging period of these wines facilitates the contact between the highly concentrated sugars and the amino acids in the medium. 5-hydroxymethylfurfural is also yielded by the wood, to the thermal degradation of hexoses and rhamnose [14,52]. Furfural, on the other hand, has its origin in the wood itself, since it is produced from the dehydration and cyclization of pentoses during the thermal treatment the casks’ wood is subjected to as part of its manufacturing process.

### 3.5. Evolution of Brown Color during Aging

The absorbance values measured were significantly different (ANOVA) depending on the aging scale. The absorbance at 470 nm allowed for the establishing of comparisons within the amber–mahogany–ebony range. The color of PXSWs increases with aging time (Table 4). This is due to hydroxylation, oxidation, and/or to the polymerization reactions of certain compounds in the wine that generally present a phenolic structure, such as quinones, among others, which change their appearance from colorless to colored [4,53]. The presence of certain metals, such as copper or iron, in wines has an influence on these oxidative processes, since they act as catalysts that facilitate the oxidation processes in PXSWs. Metal contents in PXSWs are higher than those determined in dry sherry wines of the same age [7,8]: 5–15 mg/L iron and less than 1 mg/L copper. All of the above explains the significant increments that have been registered when measuring the absorbance of these sweet sherry wines at 470 nm. Likewise, as the wines age, the transfer of certain compounds from the casks’ wood also contributes to the wines’ final color.

### 3.6. A Study on Predicting the Age of the Wines

A regression study has been proposed to determine those variables studied in the PXSW *Criaderas y Solera* system that presented a direct relationship with the age of the wine, such that a model could be developed to predict the age of the wines and to identify the most influential parameters that could facilitate wineries having a choice of routine analyses with which to control the quality of their wines.

As a first stage, a correlation study was carried out that included all of the variables in order to determine the degree of correlation between the predictive variables and age as the dependent variable. This study allowed for the removal potassium, isobutanol, and ethyl decanoate from the set of predictive variables, as they did not correlate significantly with the dependent variable, age, at a 95% confidence level. This first study also revealed that many of the remaining variables could be cross-correlated with each other, which would result in certain multicollinearity issues with respect to the final regression model.

**Figure 2:**
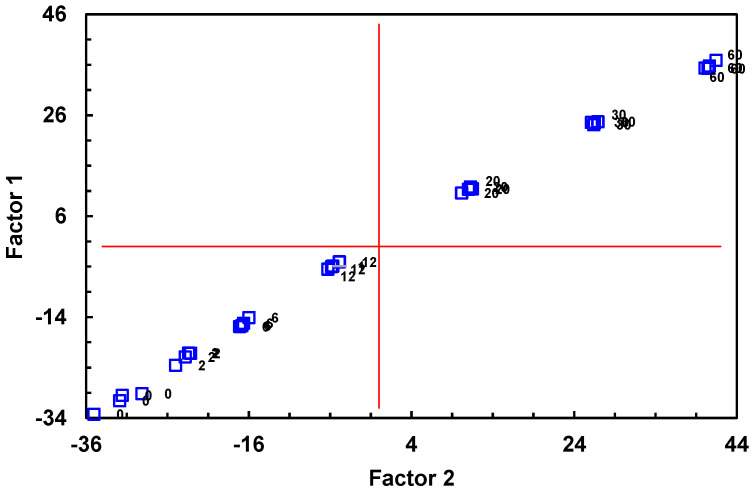
Dispersion graph (VF1 vs. VF2) of the factor analysis.

In order to identify those variables with a greater weight in a potential regression model and verify whether the data have a latent structure that could be related to the age of the wines, a factor analysis using the remaining 46 variables was then conducted. A total of 84 instances of data for each parameter was evaluated. According to this analysis, 2 factors explained 95.531% of the variability, with VF1 accounting for 93.347% of the variance. In addition, according to the dispersion graph (VF1 vs. VF2), both factors are related to the age of the wine (Figure 2).

Given this evidence, it was decided to continue the study with all of the variables whose VF1 coefficients had an absolute value equal to or greater than 0.5, which led to the removal of acetaldehyde, acetal, methanol, n-propanol, ethyl dodecanoate, calcium, n-hexanol, and ferulic acid from the set of predictive variables.

The next step consisted in searching for MLR models that would allow for the discrimination of those age-related variables that might be validated as markers or indicators of the wines’ ages.

Given the large number of remaining predictive variables (39), a strategy that aimed at generating a model with the shortest possible number of independent variables was followed. For this purpose, a maximum number of five variables was established.

As a first step, the variables were grouped by families of compounds. The family variables include total acidity, volatile acidity, sulfate, total dry extract, sugar-free extract, absorbance at 470, and FCI. A “Multiple Regression” study was used to determine the best model for each group of variables. All of the predictive variables from zero to five were combined, and the statistics for their evaluation were then calculated, including the mean square error (MSE), the R^2^ values adjusted to the degrees of freedom, Mallows’ Cp, the *p*-values of the predictive variables and that of the model at 95% significance, and the Schwarz–Bayesian information criterion (SBIC). Thus, the best model was selected according to the following criteria: *p*-values < 0.05 of the predictor and the model variables; R^2^ close to 100; and better (smaller) Mallows’ Cp as well as SBIC criteria.

Once the best model for each group of variables had been obtained, the possibility of combining the different groups of variables was verified by following the same strategy. In this case, the only potential predictive variables to be considered were the variables that had been selected for the previous models.

In the case of the PX5 model, which combined all of the families of variables, and given that the procedure that had been applied for the previous ones had presented some difficulties, these issues were probably related to multicollinearity, as it generated models with appropriate selection criteria but where the *p*-values of the variables presented a lack of significance. Therefore, and in order to overcome this issue, this new model was generated using a backward stepwise selection procedure aiming to obtain an expression of the age of the wine based on eight variables.

Table 5 presents the best models calculated for the PXSW samples. The PX5 model, which combines all of the previously selected variables, has a significance level above 95% and an R^2^ (adjusted for GL) that explains 99.992% of the variance.

In order to verify the presence of multicollinearity and also to increase the stability of the model, a ridge regression was performed. This procedure forces the model coefficients towards zero, which reduces the risk of overfitting, the variance, any effects from the correlations between predictors, and the influence of the less relevant predictors on the model.

When this procedure was applied, the PX5 II model in Table 5 was obtained, which presents an R^2^ adjusted to the degrees of freedom of 97.473 for a chain parameter of 0.065, showing variance inflation factors (VIF) coefficients below 3.

The resulting PX5 II model was influenced by the following compounds or analytical parameters: the increasing concentration with the aging of the wines of three of its most important phenolic acids, namely gallic acid, p-coumaric acid, and syringic acid, furfural, sugar-free extract, 2-phenylethanol, malic acid, and fertaric acid.

From the initial data matrix, six cases were extracted and used to validate the model after it had been built. Table 6 shows the results obtained.

### 3.7. Tasting Sessions

**Figure 3:**
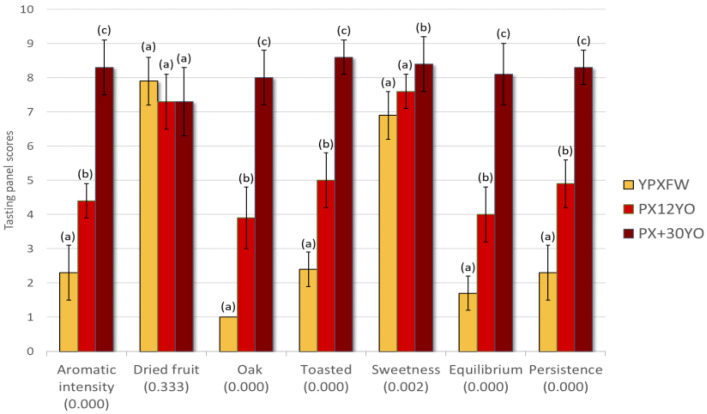
Average scores awarded by the tasting panel to the wines assessed by sensory evaluations. ANOVA: for the same descriptor, different letters indicate significant differences (*p* < 0.05).

Figure 3 contains the mean scores awarded by the panel to the samples evaluated, as well as the *p*-values resulting from the ANOVA applied to the three wines evaluated. Standard deviations of less than one for all of the wines and descriptors evaluated confirmed the high homogeneity of the panel. In general, nearly all of the descriptors (except for raisined fruit) demonstrated some discriminating capacity (*p*_anova_ < 0.05) for the evaluated wines.

The intensities corresponding to most of the olfactory and olfactory–gustatory descriptors increased after the aging of the YPXFWs, with the exception of raisined fruit, whose perception decreased somewhat (although not significantly) in comparison with the young wine. Nevertheless, it stayed above seven points in all cases. With regard to the sweetness of the wines, it was only with the over 30 years very long aging that its intensity increased to be statistically significant by more than 1 point and reached an average of 8.4 points. The rest of the PXSW descriptors increased proportionally with aging time. Thus, a clear increment in oak and toasted notes as well as a greater persistence and balance in the mouth were reported. Figure 3 allows a visual comparison of the sensory profiles of the wines evaluated.

The multivariate processing of the data via factor analysis led to similar results. The factors were extracted based on the principal components. The factor analysis was conducted by applying a varimax rotation. The factors were selected according to an eigenvalue greater than one.

**Figure 4:**
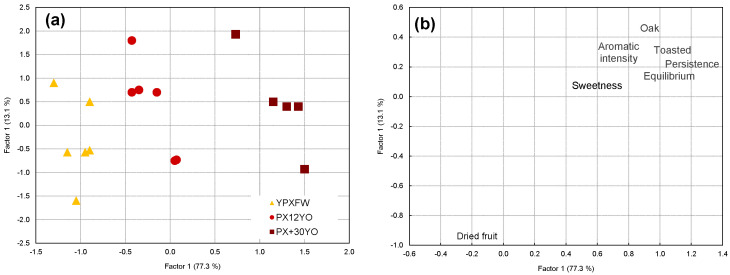
(**a**) Projection of the wines on factor 1 and 2 planes according to the factorial analysis; (**b**) weights of the sensory descriptors on factor 1 and 2 planes.

The analysis revealed 2 factors (F) that explained 90.4% of the total variability in the data (Figure 4a). F1 explains 77.3% of the variability; this factor is related to the aging time in the casks, since the ordering from left to right of the wines starts via the YPXFWs and moves through the PX12YO and up to the PX+30YO. Figure 4b shows the weights corresponding to the sensory descriptors with regard to the first two factors of the space obtained, which should allow for the confirmation of their interpretation when discriminating between the profiles of the wines evaluated. Thus, a close correlation between F1 and the oak note can be observed, as expected, but one can also be observed between aromatic intensity, toast, and persistence and balance in the mouth, all of which have correlation coefficients with an F1 value greater than 0.9. These are the descriptors that increased with the aging of the wine. On the other hand, F2 explains 13.1% of the total variability in the data; this factor presents a strong negative correlation with the aroma of raisined fruit (r < −0.9), which explains the placement of the young PX wine at slightly lower positions than those of the aged wines.

## 4. Conclusions

The characteristics of PXSWs, according to the analyses that have been conducted, are greatly influenced by the raisining processing of its grapes and by its oxidative aging. The decrease in alcohol content, due to the difficulty in maintaining the hermeticity of the casks, because of the high sugar content of the wine and the effect of the *merma*, causes many of the compounds studied to increase their concentrations during aging. Thus, an increase in the values of total acidity, volatile acidity, sulfates, glycerol, total dry extract, reducing substances, and sugar-free extract is observed as the PXSWs age. Tartaric acid content decreases with aging as it becomes insolubilized in the form of calcium and potassium salts, while the other organic acids analyzed—citric, malic, succinic, and lactic acid—increase their concentrations; of these, malic acid is the majority in the older wines. Among the volatile substances, it is worth highlighting the increase in the concentration of higher alcohols with aging, as well as esters: ethyl acetate, ethyl lactate, and diethyl succinate, which are also increased by the esterification carried out by their organic acids and the alcohol of the medium. On the contrary, a significant increase in fatty acid ethyl esters is not observed. Aging in oak barrels has shown a constant increase in FCI in all PXSWs, as well as in furanic derivatives as a consequence of Maillard reactions between sugars and nitrogenous substances in the wines. The combination of all of these factors leads to the remarkable organoleptic personality exhibited by this unique wine. With aging, the tonality in these wines changed from amber to ebony; additionally, the aromas and flavors, which in the YPXSWs were reminiscent of their origins (raisins, sweetness), changed to a greater complexity in the PXSWs that included the contribution of oak and toasted wood. This study has demonstrated the close correlation between the average age of PXSWs and the parameters that have been investigated through a multiple linear regression analysis. A model has been developed that has the capacity to determine the average age of these wines at over 97% confidence. A model of this kind should be considered as a powerful tool with which wineries can control wine quality, since it allows for the determination of a wine’s age based on just eight parameters: syringic acid, p-coumaric acid, sugar-free extract, malic acid, 2-phenylethanol, gallic acid, fertaric acid, and furfural.

## Figures and Tables

**Table 1:** Olfactory descriptors and patterns used during the work with the tasters.

Descriptor	Definition
Odor	
Aromatic intensity	Intensity of all of the positive aromatic notes of the wine.
Dried fruits	Aromas of ripe and partially dehydrated fruits due to direct exposure to sunlight (raisins, figs, dates, prunes, etc.).
Oak	Aromas of wood, with hints of vanilla and spices.
Toasted	Aromas of roasted toffee, honey, coffee, dark chocolate, liquorice, etc.
Flavor	
Sweetness	Very sweet sensation but not sickening.
Equilibrium	Good integration of the alcohol, acidity, and sugar, without astringency, but with the aromatic reminder of the oak, as appropriate for an oak-aged wine.
Persistence	Time evaluation of the olfactory–gustatory notes remaining after the final sip.

**Table 2:** Evolution of (a) general physicochemical parameters, (b) organic acids, and mineral compositions during the aging of PXSWs.

(a)	YPXFW	5th Cra.	4th Cra.	3rd Cra.	2nd Cra.	1st Cra.	Solera
Density	1139 ± 4 ^a^	1143 ± 1 ^b^	1146 ± 1 ^c^	1150 ± 1 ^d^	1154 ± 1 ^e^	1160 ± 2 ^f^	1167 ± 2 ^g^
Alcoholic strength	17.60 ± 0.13 ^a^	17.07 ± 0.08 ^b^	17.02 ± 0.10 ^b^	16.70 ± 0.05 ^c^	16.50 ± 0.10 ^d^	16.20 ± 0.10 ^e^	15.70 ± 0.05 ^f^
pH	4.50 ± 0.35 ^a^	4.45 ± 0.06 ^a^	4.41 ± 0.02 ^a,c^	4.25 ± 0.02 ^c^	4.12 ± 0.04 ^b,c^	4.07 ± 0.02 ^b^	4.06 ± 0.01 ^b^
Total acidity	2.95 ± 0.54 ^a^	3.29 ± 0.21 ^b^	3.58 ± 0.16 ^c^	4.21 ± 0.09 ^d^	4.81 ± 0.04 ^e^	5.03 ± 0.05 ^e,f^	5.25 ± 0.03 ^f^
Volatile acidity	0.52 ± 0.04 ^a^	0.56 ± 0.02 ^b^	0.62 ± 0.03 ^c^	0.68 ± 0.02 ^d^	0.77 ± 0.03 ^e^	0.92 ± 0.02 ^f^	1.06 ± 0.06 ^g^
Glycerol	2.13 ± 0.93 ^a^	3.33 ± 0.06 ^b^	3.78 ± 0.07 ^c^	4.46 ± 0.04 ^d^	6.05 ± 0.04 ^e^	7.19 ± 0.04 ^f^	9.99 ± 0.04 ^g^
**(b)**	**YPXFW**	**5th Cra.**	**4th Cra.**	**3rd Cra.**	**2nd Cra.**	**1st Cra.**	**Solera**
Tartaric ac.	3602 ± 332 ^a^	2402 ± 89 ^b^	1571 ± 99 ^c^	1299 ± 89 ^d^	961 ± 12 ^e^	876 ± 16 ^e,f^	709 ± 14 ^f^
Malic ac.	779 ± 159 ^a^	955 ± 54 ^b^	1100 ± 25 ^c^	1330 ± 47 ^d^	1753 ± 49 ^e^	1990 ± 39 ^f^	2274 ± 45 ^g^
Citric ac.	210 ± 107 ^a^	223 ± 18 ^a,b^	232 ± 7 ^a,b^	270 ± 7 ^b,c^	317 ± 11 ^c^	351 ± 7 ^c,d^	385 ± 8 ^d^
Lactic ac.	191 ± 94 ^a^	230 ± 52 ^a^	262 ± 25 ^b^	353 ± 17 ^c^	458 ± 11 ^d^	516 ± 17 ^e^	653 ± 17 ^f^
Succinic ac.	168 ± 114 ^a^	187 ± 13 ^a^	210 ± 13 ^a,b^	248 ± 14 ^b,c^	286 ± 15 ^c^	383 ± 17 ^d^	514 ± 16 ^e^
Sulfates	0.80 ± 0.24 ^a^	0.75 ± 0.04 ^a^	0.83 ± 0.04 ^a^	0.94 ± 0.04 ^b^	1.12 ± 0.03 ^c^	1.27 ± 0.03 ^d^	1.38 ± 0.04 ^d^
Calcium	80 ± 17 ^a^	76 ± 3 ^a,d^	70 ± 4 ^d^	75 ± 3 ^a,d^	86 ± 3 ^a,b^	92 ± 1 ^b,c^	99 ± 2 ^c^
Potassium	3163 ± 449 ^a^	2309 ± 63 ^d^	2489 ± 55 ^c,d^	2547 ± 10 ^b,c^	2637 ± 18 ^b,c^	2724 ± 13 ^b^	2898 ± 8 ^b^
Total SO_2_	80 ± 13 ^a^	50 ± 5 ^b^	30 ± 3 ^c^	12 ± 2 ^d^	5 ± 1 ^e^	3 ± 0 ^e^	1 ± 0 ^e^
Total dry ext.	427.8 ± 11.4 ^a^	436.7 ± 3.7 ^b^	446.5 ± 3.3 ^c^	454.3 ± 1.5 ^d^	465.6 ± 2.0 ^e^	482.4 ± 5.5 ^f^	497.4 ± 5.1 ^g^
Red. subst.	407.0 ± 11.00 ^a^	414.7 ± 3.5 ^b^	423.3 ± 3.2 ^c^	429.3 ± 1.5 ^c^	437.7 ± 2.3 ^d^	451.3 ± 5.5 ^e^	462.3 ± 5.1 ^f^
Sugar-free ext.	20.83 ± 1.86 ^a^	22.03 ± 0.31 ^b^	23.13 ± 0.12 ^c^	25.00 ± 0.10 ^d^	27.97 ± 0.29 ^e^	31.03 ± 0.40 ^f^	35.07 ± 0.29 ^g^

Mean values ± standard deviations (*n* = 4) are shown. ANOVA: for the same wine, different letters (in a line) indicate significant differences (*p* < 0.05). Cra: Criadera; ac.: acid. (a) Density (g/L); alcoholic strength (% ABV); total acidity (g TH_2_/L); volatile acidity (g AcH/L); and glycerol (g/L); (b) organic acids (mg/L); sulfates (g K_2_SO_4_/L); calcium; potassium and total SO_2_ (mg/L); total dry ext. (total dry extracts, g/L), red. subst. (reducing substances, g/L); and sugar-free ext. (sugar-free extract, g/L).

**Table 3:** Aldehydes, methanol, higher alcohols, and ethyl esters in mg/L in PXSWs.

	YPXFW	5th Cra.	4th Cra.	3rd Cra.	2nd Cra.	1st Cra.	Solera
Acetaldehyde	42.7 ± 16.8 ^a^	48.7 ± 6.0 ^a^	56.0 ± 7.0 ^b^	68.3 ± 5.5 ^c,d^	62.3 ± 2.5 ^b,c,d^	64.0 ± 5.0 ^b,c,d^	66.7 ± 4.0 ^d^
Diethyl-acetal	5.7 ± 1.5 ^a^	5.7 ± 1.2 ^a^	6.3 ± 1.5 ^a,b^	8.3 ± 1.5 ^c^	8.0 ± 1.0 ^c^	7.7 ± 0.6 ^b,c^	8.7 ± 0.6 ^c^
Methanol	159.3 ± 15.1 ^a^	143.7 ± 6.7 ^c^	138.3 ± 7.4 ^b,c^	143.3 ± 5.7 ^c^	138.7 ± 6.5 ^b,c^	130.0 ± 3.6 ^b^	135.0 ± 2.6 ^b,c^
N-Propanol	18.7 ± 4.0 ^a,b,c^	16.7 ± 3.5 ^b^	18.3 ± 1.5 ^b,c^	20.3 ± 2.5 ^a,c^	18.3 ± 1.2 ^b,c^	21.7 ± 3.5 ^a,c^	19.7 ± 1.5 ^a,b,c^
Isobutanol	11.7 ± 3.2 ^a^	10.7 ± 2.5 ^a^	7.7 ± 1.5 ^b^	7.7 ± 0.6 ^b^	10.3 ± 0.6 ^a^	11.0 ± 1.0 ^a^	11.7 ± 0.6 ^a^
2-methyl-1-butanol	2.0 ± 1.0 ^a^	2.7 ± 0.6 ^a^	2.3 ± 0.6 ^a^	4.3 ± 0.6 ^c^	5.7 ± 0.6 ^b^	6.0 ± 1.0 ^b^	7.3 ± 0.6 ^d^
3-methyl-1-butanol	9.3 ± 3.5 ^a^	12.3 ± 1.2 ^c^	10.7 ± 1.5 ^a,c^	14.3 ± 1.5 ^c^	22.0 ± 3.6 ^b^	23.7 ± 1.5 ^b,d^	25.3 ± 1.5 ^d^
Hexanol	0.22 ± 0.06 ^a^	0.29 ± 0.02 ^b^	0.26 ± 0.03 ^c^	0.25 ± 0.01 ^a,c^	0.28 ± 0.01 ^b,c^	0.30 ± 0.02 ^b,d^	0.32 ± 0.01 ^d^
2-phenylethanol	1.79 ± 1.20 ^a^	3.56 ± 0.65 ^d^	3.75 ± 0.45 ^d^	4.60 ± 0.11 ^c^	5.14 ± 0.26 ^b,c^	5.38 ± 0.48 ^b^	5.57 ± 0.23 ^b^
Ethyl acetate	42.7 ± 10.0 ^a^	57.0 ± 6.0 ^b^	56.7 ± 3.1 ^b^	63.3 ± 4.0 ^b^	108.7 ± 3.5 ^c^	121.7 ± 8.0 ^d^	129.0 ± 2.6 ^e^
Ethyl lactate	2.89 ± 1.44 ^a^	11.45 ± 1.18 ^b^	14.13 ± 0.68 ^c^	24.24 ± 0.49 ^d^	29.73 ± 0.60 ^e^	40.70 ± 1.07 ^f^	51.37 ± 1.71 ^g^
Diethyl succinate	0.35 ± 0.14 ^a^	3.02 ± 0.56 ^b^	4.94 ± 0.12 ^c^	5.74 ± 0.10 ^d^	6.78 ± 0.26 ^e^	8.29 ± 0.13 ^f^	11.17 ± 0.13 ^g^
Ethyl hexanoate	0.04 ± 0.02 ^a^	0.07 ± 0.02 ^b^	0.07 ± 0.03 ^b^	0.11 ± 0.01 ^c^	0.12 ± 0.03 ^c,e^	0.15 ± 0.03 ^d,e^	0.14 ± 0.01 ^e^
Ethyl octanoate	0.12 ± 0.01 ^a^	0.21 ± 0.05 ^b^	0.36 ± 0.03 ^c^	0.53 ± 0.04 ^d^	0.70 ± 0.07 ^e^	0.99 ± 0.05 ^f^	1.34 ± 0.07 ^g^
Ethyl decanoate	0.72 ± 0.04 ^a,c^	0.72 ± 0.10 ^a,c^	0.68 ± 0.04 ^a,b^	0.75 ± 0.03 ^c^	0.66 ± 0.02 ^b^	0.70 ± 0.03 ^a,c^	0.71 ± 0.02 ^a,c^
Ethyl dodecanoate	0.14 ± 0.05 ^a^	0.19 ± 0.02 ^b^	0.21 ± 0.02 ^c^	0.24 ± 0.02 ^d^	0.23 ± 0.01 ^c,d^	0.23 ± 0.02 ^c,d^	0.20 ± 0.01 ^e^
Ethyl tetradecanoate	0.03 ± 0.01 ^a^	0.06 ± 0.03 ^b^	0.08 ± 0.02 ^b^	0.16 ± 0.02 ^c^	0.19 ± 0.02 ^d^	0.21 ± 0.03 ^d^	0.21 ± 0.04 ^d^

Mean values ± standard deviations (*n* = 4) are shown. ANOVA: for the same wine, different letters (in a line) indicate significant differences (*p* < 0.05). YPXFW: young Pedro Ximénez fortified wine; Cra: Criadera.

**Table 4:** FCI (in mg GAE/L), phenolic and furfural compounds (in mg/L), and brown color (in unit absorbance at 470 nm) in PXSWs.

	YPXFW	5th Cra.	4th Cra.	3rd Cra.	2nd Cra.	1st Cra.	Solera
FCI	981 ± 48 ^a^	1248 ± 22 ^b^	1651 ± 23 ^c^	2034 ± 16 ^d^	2532 ± 25 ^e^	2664 ± 13 ^f^	2871 ± 8 ^g^
Caffeic ac.	8.54 ± 1.44 ^a,c^	9.63 ± 0.32 ^b,c^	8.98 ± 0.13 ^c^	7.79 ± 0.16 ^d^	5.99 ± 0.17 ^e^	4.63 ± 0.18 ^f^	3.07 ± 0.13 ^g^
p-Coumaric ac.	1.40 ± 0.39 ^a^	1.68 ± 0.04 ^b^	1.93 ± 0.06 ^c^	2.15 ± 0.08 ^c^	2.86 ± 0.09 ^d^	4.16 ± 0.15 ^e^	4.75 ± 0.19 ^f^
Ferulic ac.	0.86 ± 0.23 ^a^	0.94 ± 0.04 ^a,b^	1.02 ± 0.03 ^b^	0.96 ± 0.05 ^a,b^	0.88 ± 0.02 ^a^	0.71 ± 0.06 ^c^	0.52 ± 0.04 ^d^
Gallic ac.	7.45 ± 3.33 ^a^	9.75 ± 0.34 ^b^	13.01 ± 0.21 ^c^	15.85 ± 0.13 ^d^	21.27 ± 0.44 ^e^	28.09 ± 0.64 ^f^	34.26 ± 0.47 ^g^
p-Hydroxybenzoic ac.	3.70 ± 0.92 ^a^	4.24 ± 0.08 ^b^	4.29 ± 0.03 ^b^	4.49 ± 0.07 ^b^	6.14 ± 0.08 ^c^	8.07 ± 0.22 ^d^	10.29 ± 0.29 ^e^
Protocatechuic ac.	4.53 ± 1.25 ^a^	5.58 ± 0.11 ^b^	8.18 ± 0.13 ^c^	10.35 ± 0.44 ^d^	14.30 ± 0.22 ^e^	19.18 ± 0.67 ^f^	25.54 ± 0.27 ^g^
Syringic ac.	0.60 ± 0.13 ^a^	1.42 ± 0.07 ^b^	2.35 ± 0.07 ^c^	2.80 ± 0.11 ^d^	3.61 ± 0.10 ^e^	4.37 ± 0.06 ^f^	5.04 ± 0.09 ^g^
Vanillic ac.	0.42 ± 0.13 ^a^	0.79 ± 0.05 ^b^	1.35 ± 0.04 ^c^	1.67 ± 0.07 ^d^	2.80 ± 0.07 ^e^	3.36 ± 0.10 ^f^	4.03 ± 0.07 ^g^
Trans-Caftaric ac.	4.13 ± 0.69 ^a^	3.78 ± 0.08 ^b^	3.13 ± 0.11 ^c^	2.75 ± 0.13 ^d^	1.76 ± 0.09 ^e^	0.86 ± 0.08 ^f^	0.60 ± 0.07 ^f^
Cis-p-Coutaric ac.	2.32 ± 0.61 ^a^	2.02 ± 0.03 ^b^	1.90 ± 0.06 ^b^	1.78 ± 0.10 ^b^	1.38 ± 0.06 ^c^	0.84 ± 0.07 ^d^	0.66 ± 0.06 ^d^
Trans-p-Coutaric ac.	3.47 ± 0.69 ^a^	3.12 ± 0.06 ^b^	2.54 ± 0.04 ^c^	2.19 ± 0.15 ^d^	1.75 ± 0.04 ^e^	1.01 ± 0.08 ^f^	0.86 ± 0.07 ^f^
Fertaric ac.	2.24 ± 0.61 ^a^	1.76 ± 0.06 ^b^	1.43 ± 0.05 ^c^	1.22 ± 0.09 ^c^	0.77 ± 0.06 ^d^	0.32 ± 0.03 ^e^	0.25 ± 0.03 ^e^
p-Hydroxybenz- aldehyde	0.28 ± 0.07 ^a^	0.58 ± 0.06 ^b^	1.03 ± 0.07 ^c^	1.15 ± 0.09 ^d^	2.07 ± 0.07 ^e^	3.28 ± 0.10 ^f^	3.91 ± 0.11 ^g^
Syringaldehyde	0.64 ± 0.23 ^a^	1.12 ± 0.13 ^b^	1.79 ± 0.05 ^c^	2.15 ± 0.08 ^d^	3.65 ± 0.07 ^e^	4.74 ± 0.11 ^f^	5.65 ± 0.12 ^g^
Vanillin	0.54 ± 0.09 ^a^	0.77 ± 0.03 ^b^	1.03 ± 0.05 ^c^	1.33 ± 0.07 ^d^	2.04 ± 0.07 ^e^	2.65 ± 0.09 ^f^	2.94 ± 0.07 ^g^
Furfural	1.63 ± 0.36 ^a^	3.83 ± 0.14 ^b^	7.82 ± 0.23 ^c^	16.92 ± 0.44 ^d^	26.91 ± 0.60 ^e^	41.44 ± 0.86 ^f^	52.38 ± 0.48 ^g^
5-Hydroxymethyl- furfural	18.6 ± 1.4 ^a^	104.1 ± 6.2 ^b^	293.2 ± 5.1 ^c^	550.8 ± 6.9 ^d^	993.5 ± 7.1 ^e^	1178.8 ± 13.0 ^f^	1327.1 ± 17.1 ^g^
Brown color	1.01 ± 0.17 ^a^	2.78 ± 0.05 ^b^	4.21 ± 0.05 ^c^	10.42 ± 0.40 ^d^	12.94 ± 0.10 ^e^	14.38 ± 0.16 ^f^	16.36 ± 0.24 ^g^

Mean values ± standard deviations (*n* = 4) are shown. ANOVA: for the same wine, different letters (in a line) indicate significant differences (*p* < 0.05). YPXFW: young Pedro Ximénez fortified wine; Cra: Criadera.

**Table 5:** Regression models (MLR) to estimate the aging of Pedro Ximénez sherry wines. PX1: glycerol and organic acid model; PX2: phenolic compounds model; PX3: volatile compounds model; PX4: “other variables” model; PX5: overall model; and PX5 II: overall model corrected.

Model	Regression	R^2^ (Adjusted for DF)	*p*-Value Model (95%)
PX1	Average age (years) = −21.6212 + 0.018832 × Malic ac. (mg/L) + 0.0346286 × Succinic ac. (mg/L)	98.5422	0.0000
PX2	Average age (years) = −4.95456 + 0.0967299 × Ethyl acetate (mg/L) − 1.50828 × 2-phenylethanol (mg/L) + 0.440142 × Ethyl lactate (mg/L) + 13.7337 × Ethyl octanoate (mg/L)	99.6965	0.0000
PX3	Average age (years) = −12.3699 + 0.618877 × Gallic ac. (mg/L) + 1.33241 × Syringic ac. (mg/L) − 0.810732 × p-coumaric ac. (mg/L) + 3.24606 × Fertaric ac. (mg/L) + 0.521507 × Furfural (mg/L)	99.9546	0.0000
PX4	Average age (years) = −49.1118 + 33.7166 × Volatile acidity (g AcH/L) + 9.59436 × Sulfates (g K_2_SO_4_/L) + 1.15126 × Sugar − Free Extract (g/L)	99.4990	0.0000
PX5	Average age (years) = −17.5837 + 0.00476384 × Malic ac. (mg/L) + 0.30168 × 2-phenylethanol (mg/L) + 0.942647 × Gallic ac. (mg/L) + 0.585878 × Syringic ac. (mg/L) − 0.56033 × p-coumaric ac. (mg/L) + 4.80577 × Fertaric ac. (mg/L) + 0.362257 × Furfural (mg/L) − 0.222374 × sugar-free extract (g/L)	99.9918	0.0000
PX5 II *	Average age (years) = −19.3515 + 0.755572 × Syringic ac. (mg/L) + 2.05351 × p-coumaric ac. (mg/L) + 0.477118 × sugar-free extract (g/L) + 0.00322686 × Malic ac. (mg/L) + 0.187772 × 2-phenylethanol (mg/L) + 0.292462 × Gallic ac. (mg/L) − 0.0484612 × Fertaric ac. (mg/L) + 0.182686 × Furfural (mg/L)	97.4728	

(*) PX5 model corrected by a ridge regression for a lambda parameter of 0.065; ac.: acid.

**Table 6:** PX5 II model validation with six of the PXSW samples.

Sample	Average Age (Years)	Forecast Age (Years)	Standard Forecast Error	Absolute Error (Years)
6	2	2.9852	0.1779	0.9852
10	6	7.1515	0.1885	1.1515
14	12	11.7416	0.1823	−0.2584
18	20	20.2913	0.1865	0.2194
22	30	30.9169	0.2323	0.9169
26	40	39.0538	0.1998	−0.9462

Samples 6, 10, 14, 18, 22, and 26 were samples selected from the original data matrix not used in the MLR study.

## Data Availability

The data presented in this study are available on request from the corresponding author.

## References

[1] Durán-Guerrero E., Castro R., de Valme García-Moreno M., del Carmen Rodríguez-Dodero M., Schwarz M., Guillén-Sánchez D. (2021). Aroma of Sherry Products: A Review. Foods.

[2] Sherry Wines. https://www.sherry.wine/.

[3] European Parliament and Council of the European Union (2013). Regulation (EU) No 1308/2013 of the European Parliament and of the Council of 17 December 2013 Establishing a Common Organisation of the Markets in Agricultural Products and Repealing Council Regulations (EEC) No 922/72, (EEC) No 234/79, (EC) No 1037/2001. Off. J. Eur. Communities.

[4] Chaves M., Zea L., Moyano L., Medina M. (2007). Changes in Color and Odorant Compounds during Oxidative Aging of Pedro Ximenez Sweet Wines. J. Agric. Food Chem..

[5] Ruiz M.J., Moyano L., Zea L. (2014). Changes in Aroma Profile of Musts from Grapes Cv. Pedro Ximenez Chamber-Dried at Controlled Conditions Destined to the Production of Sweet Sherry Wine. LWT Food Sci. Technol..

[6] Herrera P., Durán-Guerrero E., Sánchez-Guillén M.M., García-Moreno M.V., Guillén D.A., Barroso C.G., Castro R. (2020). Effect of the Type of Wood Used for Ageing on the Volatile Composition of Pedro Ximénez Sweet Wine. J. Sci. Food Agric..

[7] Valcárcel-Muñoz M.J., Guerrero-Chanivet M., Rodríguez-Dodero C., García-Moreno M.D.V., Guillén-Sánchez D.A. (2022). Analytical, Chemometric and Sensorial Characterization of Oloroso and Palo Cortado Sherries during Their Ageing in the Criaderas y Solera System. Foods.

[8] Valcárcel-Muñoz M.J., Guerrero-Chanivet M., Rodríguez-Dodero M.D.C., García-Moreno M.d.V., Guillén-Sánchez D.A. (2022). Analytical and Chemometric Characterization of Fino and Amontillado Sherries during Aging in Criaderas y Solera System. Molecules.

[9] Consejo Regulador Envinado (Wine-Seasoning) Technical Specifications. https://www.sherry.wine/documents/87/especificacion_tecnica_de_envinado_rev_03.pdf.

[10] Mangas J., Rodríguez R., Moreno J., Blanco D. (1996). Volatiles in Distillates of Cider Aged in American Oak Wood. J. Agric. Food Chem..

[11] Conner J.M., Paterson A., Piggott J.R. (1992). Analysis of Lignin from Oak Casks Used for the Maturation of Scotch Whisky. J. Sci. Food Agric..

[12] Pérez-Coello M.S., González-Viñas M.A., García-Romero E., Cabezudo M.D., Sanz J. (2000). Chemical and Sensory Changes in White Wines Fermented in the Presence of Oak Chips. Int. J. Food Sci. Technol..

[13] Canas S. (2017). Phenolic Composition and Related Properties of Aged Wine Spirits: Influence of Barrel Characteristics. A Review. Beverages.

[14] Guerrero-Chanivet M., Valcárcel-Muñoz M.J., García-Moreno M.V., Guillén-Sánchez D.A. (2020). Characterization of the Aromatic and Phenolic Profile of Five Different Wood Chips Used for Ageing Spirits and Wines. Foods.

[15] OIV (2021). OIV Method OIV-MA-F1-03. Determination of the Acquired Alcoholic Strength by Volume (ASV) of Concentrated Musts (CM) and Grape Sugar (or Rectified Concentrated Musts, RCM). Compendium of International Methods of Wine and Must Analysis.

[16] (2021). OIV Method OIV-MA-AS313-01. Total Acidity. Compendium of International Methods of Wine and Must Analysis.

[17] OIV (2021). OIV Method OIV-MA-AS313-02. Volatile Acidity. Compendium of International Methods of Wine and Must Analysis.

[18] OIV (2021). OIV Method OIV-MA-AS312-05. Glycerol. Compendium of International Methods of Wine and Must Analysis.

[19] (2021). OIV Method OIV-MA-AS323-04B. Sulfur Dioxide. Compendium of International Methods of Wine and Must Analysis.

[20] OIV (2021). OIV Method OIV-MA-AS321-05A. Sulfates. Compendium of International Methods of Wine and Must Analysis.

[21] OIV (2021). OIV Method OIV-MA-AS2-03A. Total Dry Matter. Compendium of International Methods of Wine and Must Analysis.

[22] OIV (2021). OIV Method OIV-MA-AS311-01A. Reducing Substances. Compendium of International Methods of Wine and Must Analysis.

[23] Valcárcel-Muñoz M.J., Guerrero-Chanivet M., García-Moreno M.V., Rodríguez-Dodero M.C., Guillén-Sánchez D.A. (2021). Comparative Evaluation of Brandy de Jerez Aged in American Oak Barrels with Different Times of Use. Foods.

[24] OIV (2021). OIV Method OIV-MA-AS2-10. Folin-Ciocalteau Index. Compendium of International Methods of Wine and Must Analysis..

[25] Schwarz M., Rodríguez M.C., Martínez C., Bosquet V., Guillén D., Barroso C.G. (2009). Antioxidant Activity of Brandy de Jerez and Other Aged Distillates, and Correlation with Their Polyphenolic Content. Food Chem..

[26] Canas S., Caldeira I., Anjos O., Lino J., Soares A., Belchior A.P. (2016). Physicochemical and Sensory Evaluation of Wine Brandies Aged Using Oak and Chestnut Wood Simultaneously in Wooden Barrels and in Stainless Steel Tanks with Staves. Int. J. Food Sci. Technol..

[27] (2007). Sensory Analysis. General Guidance for the Design of Test Rooms.

[28] (1977). Sensory Analysis. Apparatus. Wine-Tasting Glass.

[29] (2003). Sensory Analysis—Guidelines for the Use of Quantitative Response Scales.

[30] Martínez de la Ossa E., Pérez L., Caro I. (1987). Dry Extract in Sherry and Its Evolution in the Aging of Sherry. Am. J. Enol. Vitic..

[31] Martínez de la Ossa E., Pérez L., Caro I. (1987). Variations of the Major Volatiles through Aging of Sherry. Am. J. Enol. Vitic..

[32] Carrascal García V.V. (2004). Estudio de Los Ácidos Orgánicos En Brandy de Jerez y Su Relación Con Las Prácticas Tradicionales de Elaboración. Ph.D. Thesis.

[33] Consejo Regulador de Vinos y Vinagres de Jerez El Enyesado Una Práctica Vinícola Genuinamente Jerezana, Con Más de 20 Siglos de Tradición. https://www.sherry.wine/es/noticias/el-enyesado-una-practica-vinicola-genuinamente-jerezana-con-mas-de-20-siglos-de-tradicion.

[34] Gómez-Benítez J., Grandal-Delgado M.M., Diez-Martín J. (1993). Study of the Acidification of Sherry Musts with Gypsum and Tartaric Acid. Am. J. Enol. Vitic..

[35] Ruiz H.A., Thomsen M.H., Trajano H.L. (2017). Hydrothermal Processing in Biorefineries: Production of Bioethanol and High Added-Value Compounds of Second and Third Generation Biomass.

[36] (2022). Consejería de Agricultura Pesca y Desarrollo rural Orden de 4 de Octubre de 2022, Por La Que Se Aprueban Las Solicitudes de Modificiación Normales de Los Pliegos de Condiciones de Las Denominaciones de Origen Protegidas “Jerez-Xèrés-Sherry” y “Manzanilla-Sanlúcar de Barrameda”. Boletín Of. Junta Andal..

[37] Swiegers J.H., Bartowsky E.J., Henschke P.A., Pretorious I.S. (2005). Yeast and Bacterial Modulation of Wine Aroma and Flavour. Aust. J. Grape Wine Res..

[38] Flanzy C., Andre P., Benard P., Buret M., Chambroy Y., Jouret C. (1974). Fermentation Intracellulaire Des Baies de Raisin Au Cours de Leur Métabolisme Anaérobie. Annu. Technol. Agric..

[39] Pozo-Bayón M.A., Moreno-Arribas M.V., Ronald S.J. (2011). Sherry Wines. Advances in Food and Nutrition Research.

[40] Ribéreau-Gayon P., Glories Y., Maujean A., Dubourdieu D., John Wiley & Sons Ltd. (2006). Handbook of Enology: Volume 2, The Chemistry of Wine. Stabilization and Treatments.

[41] Siener R., Seidler A., Voss S., Hesse A. (2017). Oxalate Content of Beverages. J. Food Compos. Anal..

[42] Constantini V., Bellincontro A., De Santis D., Botondi R., Mencarelli F. (2006). Metabolic Changes of Malvasia Grapes for Wine Production during Postharvest Dehydration. J. Agric. Food Chem..

[43] Bellincontro A., Nicoletti I., Valentini M., Tomas A., De Santis D., Corradini D., Mencarelli F. (2009). Integration of Nondestructive Techniques with Destructive Analyses to Study Postharvest Water Stress of Introducción 36 Winegrapes. Am. J. Enol. Vitic..

[44] Moreno Vigara J.J., Peinado Amores R.A., Ediciones A.M.V. (2010). Química Enológica.

[45] Vilela A. (2020). Non-Saccharomyces Yeasts and Organic Wines Fermentation: Implications on Human Health. Fermentation.

[46] Cacho Palomar J., de Aragón C. (2009). El Roble, La Barrica y La Crianza Del Vino Tinto.

[47] Cordero-Bueso G., Ruiz-Muñoz M., González-Moreno M., Chirino S., Bernal-Grande M.D.C., Cantoral J.M. (2018). The Microbial Diversity of Sherry Wines. Fermentation.

[48] Cernîşev S. (2017). Analysis of Lignin-Derived Phenolic Compounds and Their Transformations in Aged Wine Distillates. Food Control.

[49] Ruiz M.J., Zea L., Moyano L., Medina M. (2010). Aroma Active Compounds during the Drying of Grapes Cv. Pedro Ximenez Destined to the Production of Sweet Sherry Wine. Eur. Food Res. Technol..

[50] Moreno-Vigara J.J., García-Mauricio J.C. (2013). Pedro Ximénez and Malaga. Sweet Reinf. Fortif. Wines: Grape Biochem. Technol. Vinif..

[51] Morales F.J., Jiménez-Pérez S. (2001). Free Radical Scavenging Capacity of Maillard Reaction Products as Related to Colour and Fluorescence. Food Chem..

[52] Ruiz-Bejarano M.J., Castro-Mejías R., del Carmen Rodríguez-Dodero M., García-Barroso C. (2016). Volatile Composition of Pedro Ximénez and Muscat Sweet Sherry Wines from Sun and Chamber Dried Grapes: A Feasible Alternative to the Traditional Sun-Drying. J. Food Sci. Technol..

[53] Fabios M., Lopez-Toledano A., Mayen M., Merida J., Medina M. (2000). Phenolic Compounds and Browning in Sherry Wines Subjected to Oxidative and Biological Aging. J. Agric. Food Chem..

